# Scientific Items

**Published:** 1882-01-20

**Authors:** 


### SCIENTIFIC ITEMS.

**Is there Life in other Planets?**—This question, so often mooted of late, appears to be answered in the affirmative by the discoveries of the eminent geologist, Dr. Hahn. In meteoric stones, and especially in the class called chondrites, on account of the peculiar spherical enclosures found in them, he has recently observed an entire series of organic remains. By a laborious process of grinding down and polishing these fragments, he succeeded in producing a large number of thin laminæ or delicate stone shavings, which he subjected to a careful series of investigations under the most powerful microscopes He has recently published a book on this subject, containing on thirty-two plates more than one hundred representations of these laminæ of meteorites, every one of which contains different forms and figures, which Dr. Hahn positively identifies, not as mineralogical but as organic, and, in fact, as zoological formations belonging to the different classes of sponges, corals and crinoids. These pictures, which have been reproduced from the original laminæ by photography without any alterations or additions by a draughtsman, must cause great surprise to every geologist and palæontologist, who will at once recognize the structure of well-known coral types on several of the plates. The majority of the meteorites containing these forms are part of the celebrated great meteoric fall of Knyahinya, in Hungary, which took place on the 9th of June, 1866.—[Drug. Circular.

**To Hasten the Germination of Cotton Seed.**—A discovery of some importance has been made respecting cotton seed. Professor Thomas Taylor, who had been asked by the Agricultural Department of the United States to report on the position of the oil cells in cotton seed, found that one series was situated near the outer surface and another immediately surrounding the germinating point. Finding the seed thus protected, he experimented as to the effect of sulphuric acid of commercial strength upon the seed, and proved that while the cotton adhering to the seed was destroyed, the outer shell was not visibly affected, and that after washing and planting the seeds germinated six days earlier than seeds not so treated. This treatment facilitates the expression of the oil, and it is proposed to endeavor to remove from the sulphuric acid the glucose formed by the solution of the cotton.—[Gardeners' Chronicle.

**To Preserve Iron from Rust.**—M. Ventura Serra, after many years of experiment and observation, having noticed that knives used in cutting plants belonging to the family of Euphorbiaceæ did not rust, is led to recommend for this purpose an alcoholic solution of gum (resin of) euphorblum. This, when applied to steel, iron or copper, forms a thin, uniform, and very adherent layer, which effectually protects the metal. Experiments with copper immersed in sea-water—a ship's sheathing—were followed by gratifying results.—[Druggists' Circular.

**Tongues and Gizzards.**—A recent English writer says: The common snail sets forth to ravage our gardens equipped with 150 rows of stout serrated teeth. The whole palate contains about 21,000 teeth, while a full-grown slug has over 26,000 of these silicious spikes. The whelk has a ribbon-like tongue, contained in a probocis, with which it bores holes in the shells of the mollusks which form its food. The tongue has strong, saw-like teeth on the edges, with rows of finer ones between. In some mollusks the tongue resembles a tassellated pavement, with a tooth in the centre of each lozenge-shaped compartment,. But although the palatal system of the snails forms a most powerful and efficient apparatus for triturating their food, it more closely resembles the gizzard of birds than the teeth of quadrupeds, and it is in the class of fishes that we find the first examples of true teeth, set in a bony socket and ranged at the opening of the alimentary canal. At what time the fashion of wearing teeth came in we have no means of ascertaining. If, however, the Darwinian theory be correct, at some enormously remote period of time some lucky animal developed the new weapon by a series of fortunate variations, and its possession gave to him and his posterity such a "pull" over their competitors that they were able to set the fashion, which has lasted to the present day.—[Jour. of Chem.

**A New Gaseous Disinfectant.**—M. Peyrusson has communicated to the Academie des Sciences the result of a series of experiments made with the fumes of nitrous ether. From these it has been demonstrated that they possess a most manifest disinfectant and anti-putrid action.

In addition to the trials made with it in the laboratory, it has been employed in the hospitals of Limoges. This agent has successfully purified the wards. It has also been used to preserve cadavers.

This ether offers another advantage: Its odor is mild, agreeable and entirely inoffensive.—[Monthly Review of Medicine and Surgery.

**What is Sulphur?**—Less than a hundred years ago this question was answered by Stahl as follows: Sulphur consists of oil of vitriol (sulphuric acid) combined with combustible essence. Sulphur can be made out of sulphuric acid, he said, by heating it with charcoal dust, because the latter contains the combustible essence or being. But this combustible essence had not been isolated and obtained in a free state, which seemed to puzzle some of the skeptics of that day, and led them to deny the existence of oil of vitriol in the sulphur.—[Ibid.

**Cosmical Hail.**—M. Shevedoff, a Russian scientist, advances the remarkable hypothesis that hail is of cosmical origin, and captured by our world in its journey through space in the same manner that meteorites are attracted to our planet. He does not, however, explain why the heat generated when the hail-stones strike our atmosphere does not melt them.—[Ibid.

